# Predictive Factors for Early Initiation of Artificial Feeding in Patients With Sporadic Creutzfeldt-Jakob Disease

**DOI:** 10.3389/fneur.2018.00496

**Published:** 2018-07-03

**Authors:** Pei-Chen Hsieh, Han-Tao Li, Chun-Wei Chang, Yih-Ru Wu, Hung-Chou Kuo

**Affiliations:** Department of Neurology, Linkou Chang Gung Memorial Hospital, College of Medicine, Chang Gung University, Taipei, Taiwan

**Keywords:** creutzfeldt-jakob disease, artificial feeding, pognosis, myoclonus, akinetic mutism

## Abstract

**Background:** Akinetic mutism has often been used as the predictor of sporadic Creutzfeldt-Jacob disease (sCJD) endpoints, but it may be difficult for general physcians to assess. Nasogastric (NG) tube insertion is indicated for many neurodegenerative diseases with a clinical course of swallowing failure, and can be more easily identified than akinetic mutism by general physicians. Therefore, the aim of this study was to identify whether there are predictive factors for early initiation of artificial feeding in patients with sCJD who require enteral nutrition due to swallowing failure.

**Methods:** We retrospectively reviewed the medical records of all patients diagnosed with probable sCJD who were admitted to the neurology ward at a medical center in Taiwan from January 2002 to July 2017. We used Pearson's chi-squared test to detect the correlation of initial symptoms, neurological signs, brain magnetic resonance imaging (MRI), electroencephalography (EEG), and increased levels of 14-3-3 protein in cerebrospinal fluid (CSF) analysis. The Cox proportional hazards model was used to detect prognostic factors for early initiation of NG tube insertion in sCJD patients.

**Results:** The onset age ranged from 51 to 83 years, and mostly ranged from 60 to 79 years. Akinetic mutism was correlated with pyramidal tract signs, myoclonus, and extrapyramidal signs. Furthermore, myoclonus was revealed to be associated with pyramidal tract signs. Multivariate Cox regression analysis showed that myoclonus and elevated CSF levels of 14-3-3 protein are predictive of early NG insertion.

**Conclusions:** Increased levels of 14-3-3 protein in CSF and the presence of myoclonus at diagnosis are predictive of early swallowing difficulty and indicate rapid deterioration in probable sCJD. In addition to akinetic mutism, early initiation of artificial feeding can be used to predict early deterioration in sCJD.

## Introduction

The annual global incidence rate of sporadic Creutzfeldt-Jakob disease (sCJD) is approximately 1–1.5 cases per million people (1). In Taiwan, the incidence rate is 0.55 cases per million people per year. The pathogenesis of sCJD remains unclear. There are variable clinical symptoms and signs in sCJD. The median survival duration for patients with sCJD is 4–6 months (2, 3), but Asian populations may have longer survival (4, 5). Longer survival has been associated with younger age, female gender, absence of CSF 14-3-3 protein and type 2a prion protein, sharp-wave complexes on EEG, and MV form in codon 129 PRNP human gene (6, 7). Survival has been found to be prolonged in patients who received tube feeding after they reached the state of akinetic mutism (8).

Our patients were genotyped at codon 129 of the PRNP gene and all were methionine homozygotes (MM) from 1998 to 2007, and the type was referred to as myoclonic type of sCJD (5, 9). sCJD is a rapidly progressive dementia that leads to decreased level of consciousness and cortical impairment which causes unsafe swallowing. The use of a nasogastric (NG) tube is a risk factor for mortality in advanced dementia (10–12). The initiation of artificial feeding indicates that patients have lost the ability to perform activities of daily living (ADL) and the disease has progressed to the final stage (13, 14). Although other studies have used akinetic mutism as the predictor for sCJD endpoints, it is difficult for general physicians to assess in patients whose consciousness level is impaired (4, 15, 16). Dysphagia is used for prognosis in spinal and bulbar muscular atrophy but there is a lack of reliable clinical markers for dysphagia assessment (17–19). The requirement for initiation of feeding tube insertion is easier to identify than detection of swallowing failure by a general physician or even a caregiver.

Although tube feeding may prolong survival in sCJD, the initiation time of artificial feeding may be a more suitable endpoint than akinetic mutism. Therefore, the goal of this study was to determine if there are predictive factors for early NG tube feeding.

## Methods

We retrospectively reviewed the medical charts of all patients diagnosed with probable sCJD and admitted to the neurology ward from January 2002 to July 2017 at Chang Gung Memorial Hospital. All of the cases were submitted by physicians who diagnose sCJD and were reviewed by the neurology, neuroradiology, and neuropathology experts of the CJD Surveillance Unit (CJDSU) at the Taiwan Neurological Society. We had a total of 35 probable sCJD patients. We included patients whose initial symptoms occurred within 2 months of admission and who could have a neurological examination with a Glasgow coma scale (GCS) score of 15 out of 15. We excluded a patient who had a history of progressive supranuclear palsy that made it difficult to identify initial neurological symptoms. Data were collected at baseline included gender, age, time of admission, cerebrospinal fluid (CSF) with 14-3-3 proteins, presence of high signal on diffusion-weighted images of magnetic resonance imaging (MRI), periodic sharp wave complex (PSWC) in electroencephalography (EEG) findings, NG tube insertion time, initial presentation, and abnormal neurological examination at admission. Results of CSF 14-3-3 protein assay data were obtained through collaboration with the Taiwan Prion Disease Pathology Surveillance Center. Clinical signs and symptoms that were assessed by neurologists included cognitive impairment (memory impairment, visual spatial dysfunction, apraxia, aphasia, and akinetic mutism), visual disturbance (visual field defect, visual hallucination, complex visual disturbance), cerebellar disturbance (ataxic gait, limbs dysmetria), pyramidal dysfunction (hemiparesis, spasticity, hyperreflexia, positive Babinski sign), extrapyramidal dysfunction (rigidity, bradykinesia, dystonia, dyskinesia), myoclonus, and seizure. NG tube insertion was performed when a patient was assessed by a clinical physician as having decreased level of consciousness or severe cognitive impairment that caused poor intake. The date of NG insertion was recorded according to medical record. The protocol was approved by the institutional review board of Chang Gung Memorial Hospital (IRB No.: 201701320B0).

### Statistical analysis

The time from admission to NG insertion of all patients were compared with age, sex, neurological signs, the elevation of CSF 14-3-3 protein, and brain MRI findings. Four patients did not have the records of NG insertion times because of loss to follow-up. We used Pearson's test was used to estimate the correlation of initial symptoms, clinical neurological signs, MRI image findings, and 14-3-3 proteins in CSF. For the cumulative incidence of NG tube insertion, the Kaplan–Meier method was used for overall analysis and each of the above features was stratified. The Cox proportional hazards model was used to identify the prognostic factors for the duration from onset to NG tube dependence, and the backward selection method in multivariate analysis was used to assess the independent effects of the investigated factors. Statistical significance was selected using the selection method at *P* < 0.05 in univariate regression analyses. We used the Wald test to estimate hazard ratios (HRs) and 95% confidence intervals (CIs), and we used Pearson's chi-squared test to detect the correlation of neurological signs at admission, imaging findings, 14-3-3 proteins in CSF, and initial PSWC on EEG. Statistical analyses were performed using SPSS version 22.

## Results

**Figure 1:**
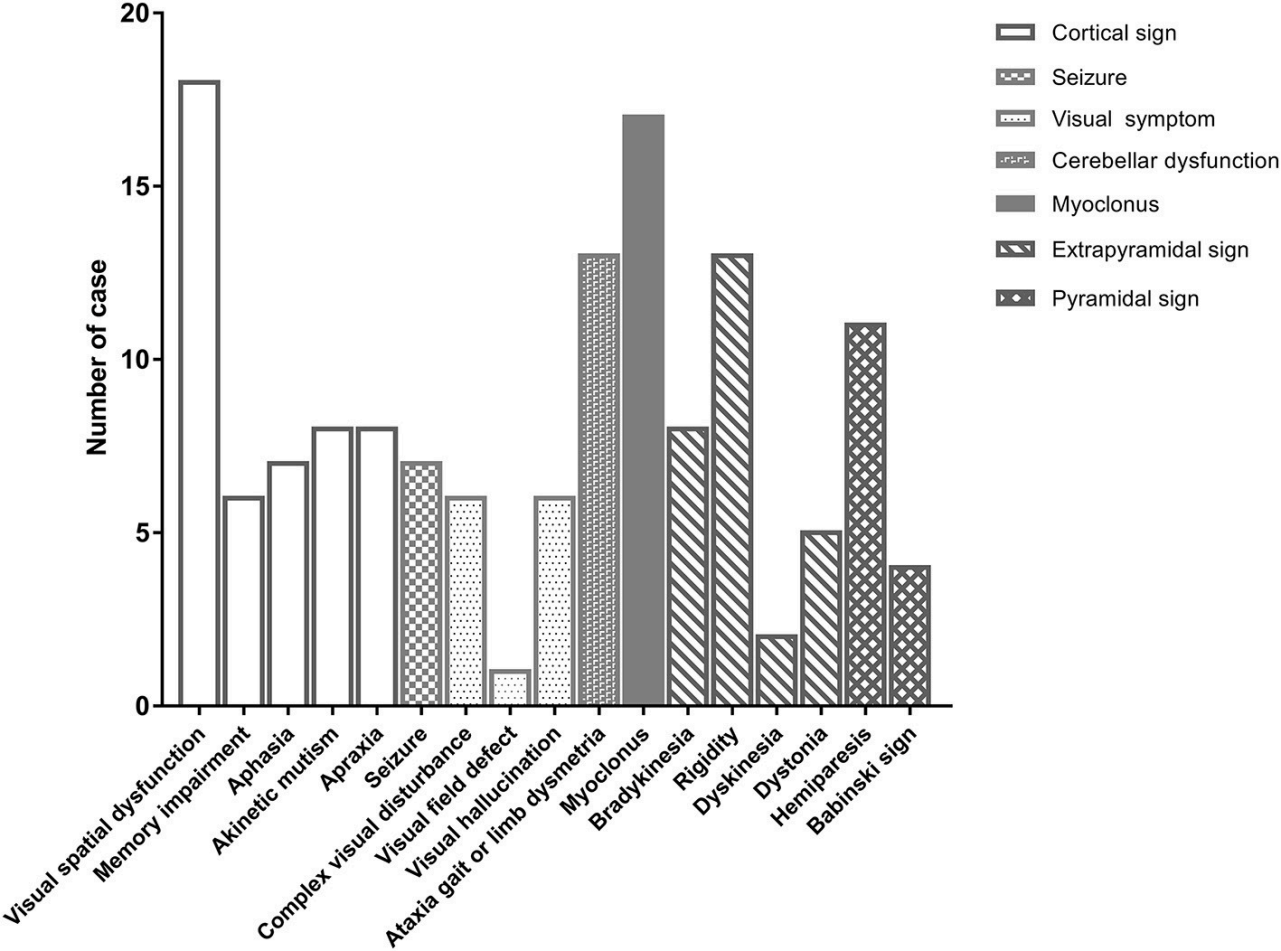
The initial neurological signs of patients with sporadic Creutzfeldt-Jakob disease at first hospitalization.

Thirty patients with probable sCJD admitted from January 2002 to July 2017 were included in the study. Basic characteristics are summarized in Table 1. The onset age ranged from 51 to 83 years, and mostly ranged from 60 to 79 years. The median age at diagnosis was 68.5 years. Female patients accounted for 43% of the patients in this study. Increased levels of CSF 14-3-3 protein were found in 22 of 30 patients. The PSWC of initial EEG was 33%. Among all patients, 47% had the characteristic brain MRI findings indicating cortical ribbon signs and 53% had the MRI findings indicating both cortical and basal ganglia involvement (Table 1). The initial symptoms of cognitive impairment, visual disturbance, unsteadiness, and speech disturbance were present in 43, 37, 37, and 27% of the patients, respectively. The most common neurological sign during admission was visual spatial dysfunction, which was followed by myoclonus and cerebellar and extrapyramidal dysfunction (Figure 1).

**Table 1:** Baseline demographic and clinical characteristics of sCJD patients.

**Patients with sCJD**	***n* = 30**	**(%)**
**SEX**		
Female	12	43
Male	18	57
**Age of Onset ± SD (year)**	67.7 ± 9.3	
Median (year)	68.5	
Range (year)	51-83	
**AGE AT DIAGNOSIS (YEAR)**		
40–49	0	
50–59	6	20
60–69	12	40
70–79	9	28
80–89	3	10
**Initial symptom to admission ± SD (day)**	27.5 ± 12	
**CLINICAL MANIFESTATIONS AT DISEASE ONSET (%)**		
Cognitive impairment	10	33
Visual disturbance	11	37
Gait disturbance	11	37
Speech disturbance	8	27
**Increased 14-3-3 protein in CSF (%)**	22/30	73
**PSWC in Initial EEG (%)**	10/30	33
**BRAIN MRI FINDINGS**		
Cortical involvement	14/30	47
Cortical and basal ganglia involvement	16/30	53

*CSF, Cerebrospinal fluid; EEG, electroencephalography; MRI, magnetic resonance imaging; PSWC, periodic sharp wave complex; sCJD, sporadic Creutzfeldt-Jakob disease*.

### Correlations among variables at diagnosis

**Figure 2:**
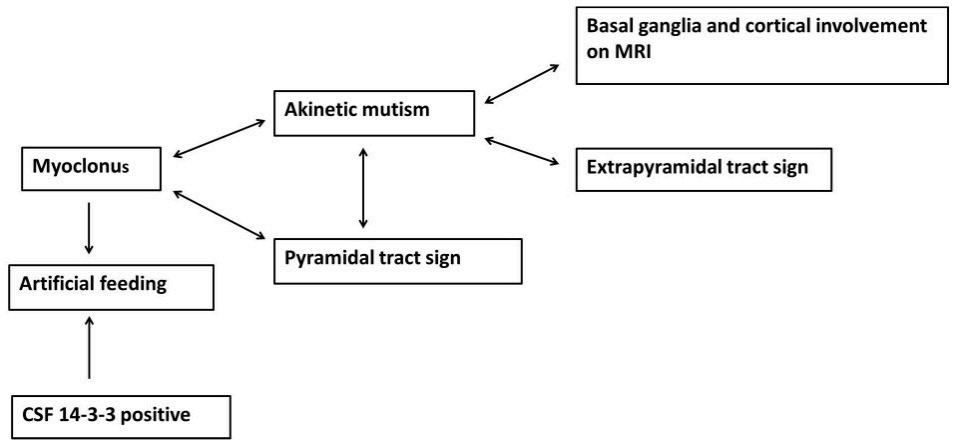
The association between neurological signs and brain MRI findings as well as myoclonus and detectable 14-3-3 protein in CSF leading to early artificial feeding in sporadic Creutzfeldt-Jakob disease.

The Pearson's x^2^ test was used to assess correlation between neurological signs and variables, and the variables included basal ganglia and cortical involvement on brain MRI, PSWC on initial EEG, increased 14-3-3 protein in CSF, sex, and age. In addition, the neurological signs were also compared with each other. Both basal ganglia and cortical involvement on brain MRI were significantly correlated with akinetic mutism (*P* < 0.01). Akinetic mutism was correlated with abnormal pyramidal and extrapyramidal signs and myoclonus. Furthermore, myoclonus was associated with pyramidal tract signs. Figure 2 shows correlations between investigative tests and neurological signs and between variables at diagnosis. An association was revealed between myoclonus, akinetic mutism, and pyramidal tract dysfunction. Furthermore, myoclonus and detectable CSF 14-3-3 protein might lead to early use of artificial feeding.

### Relationships between clinical signs and symptoms and NG tube insertion time

**Figure 3:**
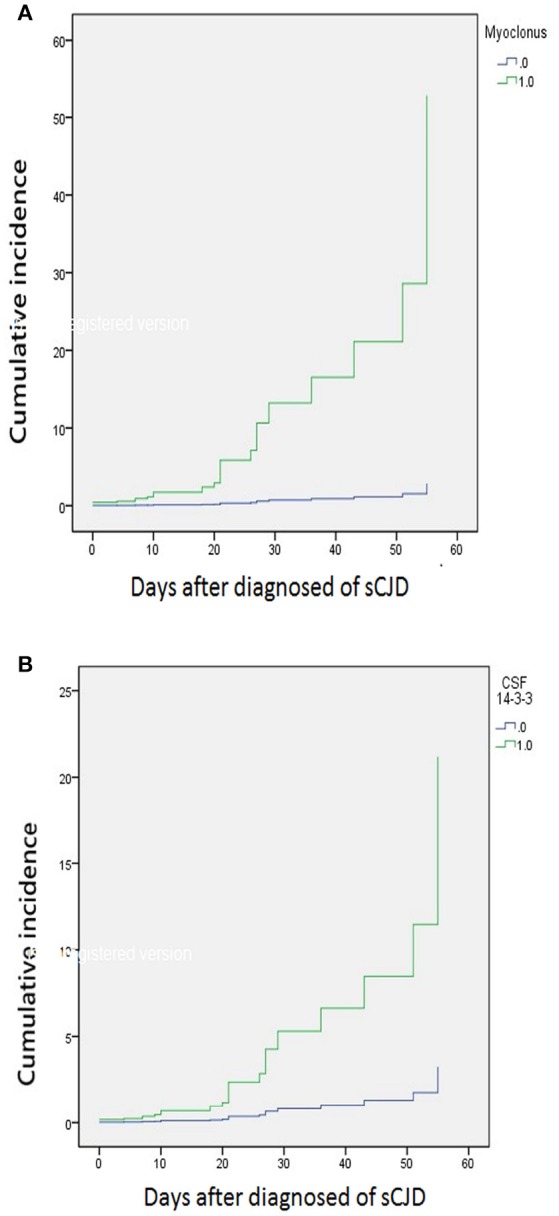
Stratified cumulative incidence of artificial feeding in **(A)** myoclonus and **(B)** 14-3-3 protein in CSF at diagnosis of sporadic Creutzfeldt-Jakob disease.

The median time of NG tube insertion was 46.4 days after the onset of the initial symptom. Table 2 shows a univariate Cox regression analysis of gender, age, PSWC in EEG, MRI with both basal ganglia and cortical involvement, elevation of CSF 14-3-3 protein, and neurological signs during initial hospitalization. The result demonstrated significant differences in the CSF 14-3-3 protein, myoclonus, pyramidal dysfunction, extrapyramidal dysfunction, and akinetic mutism. These were attributed to NG tube insertion. Patients with increased 14-3-3 protein in CSF were found to have 6.5 times higher risk of early NG tube insertion. No significant differences were found in the characteristic findings of sCJD on EEG and brain MRI with both cortex and basal ganglia involvement. The median periods from first hospitalization to NG tube insertion in myoclonus and CSF analysis were 10 and 19 days, respectively. Figure 3 shows the cumulative incidences of artificial feeding in sCJD patients with (3A) myoclonus and (3B) 14-3-3 protein in CSF.

**Table 2:** Univariate and multivariate regression analyses of the day of nasogastric tube insertion.

**Variables**	**HR**	**Univariate *P*-value**	**95% CI**		**HR**	**Multivariate *P*-value**	**95% CI**	
			**Lower**	**Upper**			**Lower**	**Upper**
Gender: male	1.29	0.539	0.574	2.887				
Age at diagnosis > 65 (years)	1.40	0.426	0.613	3.180				
PWSC in EEG	1.76	0.203	0.738	4.181				
Cortical and basal ganglia involvement	1.77	0.188	0.767	4.117				
Increase in CSF 14-3-3 protein	2.85	0.039^**^	1.056	7.669	6.529	0.003^**^	1.928	22.109
**CLINICAL SIGNS**								
Myoclonus	5.61	0.001^**^	2.027	15.511	18.51	< 0.001^**^	4.875	70.279
Cognitive dysfunction	0.55	0.430	0.126	2.417				
Pyramidal dysfunction	4.08	0.007^**^	1.466	11.356				
Extrapyramidal dysfunction	2.72	0.029^*^	1.109	6.658				
Cerebellar sign	1.07	0.868	0.474	2.419				
Cortical visual dysfunction	0.98	0.953	0.423	2.249				
Akinetic mutism	4.19	0.009^**^	1.438	12.215				
Seizure	1.73	0.287	0.632	4.706				

EEG, electroencephalography; MRI, magnetic resonance imaging; PSWC, periodic sharp wave complex;

* *p < 0.05*,

** *p < 0.01*.

## Discussion

Swallowing requires coordination of multiple bulbar muscles and relatively clear consciousness (14). NG tube insertion is globally used for many neurological patients who suffer from swallowing failure and poor nutrition, and swallowing failure indicates that the patient's condition has reached the end of life in many neurodegenerative diseases (13). Therefore, NG tube feeding is a predictive factor of mortality in advanced dementia (12). On the other hand, feeding tube insertion increases the survival of CJD patients due to better supportive care (8).

Creutzfeldt-Jacob disease is a prion disease that can present as irreversible rapidly progressive dementia, accompanied by myoclonus, pyramidal tract, extrapyramidal tract, or cerebellar involvement (20). Most of the patients with decreased level of consciousness or severe cognitive dysfunction need NG tube feeding for nutrition support during disease progression. In Chinese culture and Taiwan's health insurance system, the majority of patients receive intensive life-sustaining treatment, and some even continue the treatment until there is irreversible advanced neurological disorder. Unlike akinetic mutism, NG tube insertion makes it easier for clinical physicians to detect patients with severe neurodegenerative disease. Furthermore, the time until death of patients with NG tube feeding can be influenced by underlying comorbidities such as aspiration pneumonia or other infectious diseases. NG tube insertion caused by swallowing failure is an early sign of poor outcome in patients with dementia syndromes. Increased 14-3-3 protein in CSF has been considered as an indicator of rapid neuronal damage (7). Our study found that the presence of myoclonus and elevated levels of CSF 14-3-3 protein led to a rapid deterioration of the patient's condition and the consequent need for enteral nutrition. According to Nakatani et al. akinetic mutism is the neurological symptom of sCJD endpoint. Cerebellar disturbance and psychiatric symptoms increase the risk of akinetic mutism. Myoclonus is a potential contributing factor of akinetic mutism and exercise-induced seizures, and myoclonus and myoclonic seizures are associated with pyramidal dysfunction (16). In our study, akinetic mutism was associated with both cortical and basal ganglia involvement on brain MRI, pyramidal dysfunction, myoclonus, and extrapyramidal signs, indicating that neocortex and subcortical structures may be severely involved when akinetic mutism occurs. Therefore, since myoclonus, akinetic mutism, and pyramidal tract dysfunction are closely related, we may predict that the risk of swallowing failure increases when a patient presents with myoclonus.

Myoclonus in sCJD can present as irregular or rhythmic positive and negative jerky movements (21–23). It occurs in 82–100% of CJD patients and can be a focal or generalized pattern during the disease course, especially in the majority of advanced forms of all genotypes (24, 25). The presence of myoclonus has been considered to originate in the brainstem or thalamus area (15). Periodic rhythmic synchronized myoclonus has been suspected due to a hyperexcitable cortico-subcortical loop (26, 27). Furthermore, myoclonus occurred more frequently and earlier in MM type at codon 129 of the prion protein gene compared to other codon 129 polymorphism (9, 28). This genotype was considered to have the shortest survival compared to MV and VV forms of the human PRNP gene (7, 9). According to Iwasaki et al. CJD patients who have early onset of myoclonus, rapid disease progression to the akinetic mutism state, and PSWC on EEG were associated with relatively poor prognosis (8).

In conclusion, the dependence on artificial feeding could be a time point to predict the prognosis for CJD patients. The presence of myoclonus in CJD patients increases the risk of the need for artificial feeding and indicates an early irreversible dependence in daily activities.

## Author contributions

P-CH wrote the initial manuscript and statistical analysis. H-TL collected data of all of the sCJD patients. P-CH, C-WC, Y-RW, and H-CK involve in conception and design and analysis and interpretation of data. H-CK also gave revision of manuscript for important intellectual content.

### Conflict of interest statement

The authors declare that the research was conducted in the absence of any commercial or financial relationships that could be construed as a potential conflict of interest. The reviewer CH and handling Editor declared their shared affiliation.
